# Clinical Characteristics of Patients with Tick-Borne Encephalitis (TBE): A European Multicentre Study from 2010 to 2017

**DOI:** 10.3390/microorganisms9071420

**Published:** 2021-06-30

**Authors:** Benno Kohlmaier, Nina A. Schweintzger, Manfred G. Sagmeister, Vendula Švendová, Daniela S. Kohlfürst, Astrid Sonnleitner, Manuel Leitner, Andrea Berghold, Erich Schmiedberger, Franz Fazekas, Alexander Pichler, Jana Rejc-Marko, Daniel Růžek, Lucie Dufková, Darina Čejková, Petr Husa, Martina Pýchová, Lenka Krbková, Václav Chmelík, Věra Štruncová, Dace Zavadska, Guntis Karelis, Aukse Mickiene, Joanna Zajkowska, Petra Bogovič, Franc Strle, Werner Zenz, the EU-TICK-BO STUDY GROUP

**Affiliations:** 1 Department of General Paediatrics, Medical University of Graz, 8036 Graz, Austria; benno.kohlmaier@medunigraz.at (B.K.); nina.schweintzger@medunigraz.at (N.A.S.); manfred.sagmeister@medunigraz.at (M.G.S.); svendula@gmail.com (V.Š.); daniela.kohlfuerst@medunigraz.at (D.S.K.); astrid.sonnleitner@uniklinikum.kages.at (A.S.); manuel.leitner@medunigraz.at (M.L.); 2 Institute for Medical Informatics, Statistics and Documentation, Medical University of Graz, 8036 Graz, Austria; andrea.berghold@medunigraz.at (A.B.); erich.schmiedberger@medunigraz.at (E.S.); 3 Department of Neurology, Medical University of Graz, 8036 Graz, Austria; siegrid.fuchs@medunigraz.a (F.F.); alexander.pichler@medunigraz.at (A.P.); 4 Department of Infectious Diseases and Febrile Conditions, University Clinical Center Maribor, 2000 Maribor, Slovenia; janaa.marko@gmail.com; 5 Biology Centre of the Czech Academy of Sciences, Institute of Parasitology, Ceske Budejovice, 370 01 Ceske Budejovice, Czech Republic; ruzekd@paru.cas.cz; 6 Veterinary Research Institute, 621 00 Brno, Czech Republic; lucie.dufkova@tzpro.cz (L.D.); cejkovad@feec.vutbr.cz (D.Č.); 7 Deptartment of Infectious Diseases, University Hospital Brno, 625 00 Brno, Czech Republic; husa.petr@fnbrno.cz (P.H.); pychova.martina@fnbrno.cz (M.P.); 8 Department of Infectious Diseases, Faculty of Medicine, Masaryk University, Kamenice 753/5, 625 00 Brno, Czech Republic; 9 Department of Children’s Infectious Disease, Faculty of Medicine and University Hospital, Masaryk University, 613 00 Brno, Czech Republic; krbkova.lenka@fnbrno.cz; 10 Department of Infectious Diseases, Hospital Ceske Budejovice, 370 01 Ceske Budejovice, Czech Republic; chmelik@nemcb.cz; 11 Department of Infectious Disease and Travel Medicine, University Hospital Plzeň and Faculty of Medicine in Pilsen, Charles University, 306 05 Plzeň, Czech Republic; struncova@fnplzen.cz; 12 Department of Paediatrics, Children Clinical University Hospital, Riga Stradins University, LV-1004 Riga, Latvia; dzavadska@apollo.lv; 13 Department of Neurology and Neurosurgery, Riga East University Hospital, Riga Stradins University, LV-1004 Riga, Latvia; guntis.karelis@gmail.com; 14 Department of Infectious Diseases, Lithuanian University of Health Sciences, 44307 Kaunas, Lithuania; amickiene@gmail.com; 15 Department of Infectious Diseases and Neuroinfections, Medical University Bialystok, 15-089 Bialystok, Poland; zajkowsk@umb.edu.pl; 16 Department of Infectious Diseases, University Medical Centre Ljubljana, 1000 Ljubljana, Slovenia; petra.bogovic@kclj.si (P.B.); franc.strle@kclj.si (F.S.)

**Keywords:** tick-borne encephalitis, vaccine-preventable disease, meningomyelitis, central paresis, peripheral paresis

## Abstract

Tick-borne encephalitis (TBE) virus is a major cause of central nervous system infections in endemic countries. Here, we present clinical and laboratory characteristics of a large international cohort of patients with confirmed TBE using a uniform clinical protocol. Patients were recruited in eight centers from six European countries between 2010 and 2017. A detailed description of clinical signs and symptoms was recorded. The obtained information enabled a reliable classification in 553 of 555 patients: 207 (37.3%) had meningitis, 273 (49.2%) meningoencephalitis, 15 (2.7%) meningomyelitis, and 58 (10.5%) meningoencephalomyelitis; 41 (7.4%) patients had a peripheral paresis of extremities, 13 (2.3%) a central paresis of extremities, and 25 (4.5%) had single or multiple cranial nerve palsies. Five (0.9%) patients died during acute illness. Outcome at discharge was recorded in 298 patients. Of 176 (59.1%) patients with incomplete recovery, 80 (27%) displayed persisting symptoms or signs without recovery expectation. This study provides further evidence that TBE is a severe disease with a large proportion of patients with incomplete recovery. We suggest monitoring TBE in endemic European countries using a uniform protocol to record the full clinical spectrum of the disease.

## 1. Introduction

Tick-borne encephalitis (TBE) is an infection of the central nervous system (CNS) caused by the tick-borne encephalitis virus (TBEV) being transmitted by ticks in several central, eastern, and northern European countries [1,2]. The severity of the disease is broad, ranging from fever and headache to death, with a relatively high proportion of patients needing intensive care unit (ICU) treatment. Most patients develop meningitis or meningoencephalitis, some present with additional spinal involvement. At hospital discharge, many patients suffer persisting signs like ataxia and tremor; symptoms such as headache or decreased concentration are also described [3,4,5]. In addition, follow-up studies have shown that 16–50% of patients suffer from long-lasting sequelae [6,7,8,9,10,11]. Since the 1970s, a highly effective vaccine against TBE has been available and has led to a significant decrease in cases in countries with high vaccination rates [12]. Nevertheless, TBE remains an important issue caused by climate change and residual low vaccination rates in several endemic countries [13,14]. Therefore, continuous monitoring and detailed clinical analysis are needed to inform health care professionals and public authorities. To the best of our knowledge, hitherto, only national or single-center studies describing clinical details of TBE have been published. Here, we present a detailed description of clinical characteristics of a large international cohort of patients fulfilling the European Centre for Disease Prevention and Control (ECDC) case definition for confirmed TBE by using a common clinical protocol.

## 2. Materials and Methods

### 2.1. Establishment of a Uniform Case Record Form (CRF) and Patient Recruitment

In 2013, the international network “European genetics study of tick-borne encephalitis” (EU-TICK-BO) was established to investigate host genetic associations with the susceptibility and severity of TBE. To provide a consistent description of TBE patients across all centers, a uniform patient case record form (CRF) was established (Supplementary CRF). Paper CRFs were filled out by the treating clinicians and entered into an electronic database. In participating centers (Table S1), patients were prospectively collected between January 2014 and December 2017. A retrospective cohort was collected, starting from January 2010. We used the ECDC case definition for TBE [1]. Clinical criteria included any person with symptoms or signs of inflammation of the CNS (e.g., meningitis, meningoencephalitis, meningoencephalomyelitis). Laboratory criteria included at least one of the following: specific IgM and IgG antibodies in blood; specific IgM antibodies in cerebrospinal fluid (CSF); seroconversion or four-fold increase of TBE-specific IgG antibodies in paired serum samples. We included only patients with confirmed TBE, meeting the clinical and laboratory criteria. Patients with a history of TBE vaccination were enrolled if infection with TBEV was demonstrated either by an increase of TBEV IgG antibodies in convalescent serum, by demonstration of TBEV-IgM in CSF, or by proof of a positive TBE-specific IgG antibody CSF/serum index [15].

### 2.2. Quality Control

Data integrity and plausibility were reviewed by two clinicians from the lead study center. A list of missing items and unclear issues was sent out to each participating center. If the centers were not able to provide a complete basic dataset (age, gender, admission date, reliable laboratory evidence of a recent TBEV infection, and symptoms/signs in acute stage), their patients were excluded.

### 2.3. Patient Assessment

The findings of each patient in the neurological phase were reviewed, and the following diagnoses were assigned (modified according to Günther et al. 1997) [16]: meningitis, moderate meningoencephalitis, severe meningoencephalitis, meningomyelitis, meningoencephalomyelitis with moderate encephalitis, and meningoencephalomyelitis with severe encephalitis. The following signs indicated moderate encephalitis: Glasgow Coma Score (GCS) ≥ 9, ataxia, tremor, dysphagia, or single cranial nerve palsy. Severe encephalitis was characterized by GCS < 9, seizures, central paresis, mechanical ventilation, multiple cranial nerve palsies, or bulbar symptoms.

Pareses of extremities were categorized as peripheral paresis (a patient with flaccid paresis of an extremity/extremities without signs of corresponding lesions in brain imaging, if available) or central paresis (a patient with a spastic paresis of extremities, with signs of corresponding lesions in brain imaging, if available). At discharge from hospital, patients were categorized into “complete recovery” if symptoms or signs of TBE completely vanished or “incomplete recovery at discharge” when TBE symptoms or signs were still present. Patients with “incomplete recovery at discharge” were further categorized into “complete recovery expected” and “incomplete recovery expected” based on the assessment of the treating physician. Persisting symptoms or signs at discharge were categorized into “mild subjective” if patients had one or two subjective symptoms, “severe subjective” with three or more subjective symptoms, and “objective” if at least one objective sign with or without subjective symptoms was observed. The main foci of this work were “findings in acute disease”, “outcome at discharge”, and “blood and CSF findings”. Only “findings in acute disease” were recorded in all included patients.

### 2.4. Statistical Analysis

Differences in patient characteristics in acute disease and outcome at discharge were analyzed with the Kruskal–Wallis test with Dunn’s multiple comparisons test for continuous variables and Fisher’s exact or Pearson’s chi-squared test for categorical variables. The Kruskal–Wallis test, with Dunn’s multiple comparisons test, was performed to examine the differences in blood and CSF levels according to diagnostic groups. For binary data, *p*-values (*p*), together with odds ratios (OR) and their confidence intervals (CI), were reported. Statistical analyses were conducted in R version 3.6.0 (R Foundation for Statistical Computing, Vienna, Austria) and GraphPad Prism software version 8 (GraphPad Software, La Jolla, CA, USA).

### 2.5. Literature Review

To set our findings in context with previously published TBE cohort descriptions, a literature search was conducted on PubMed using the search term “(tick borne encephalitis [Title/Abstract])”. Titles were screened for cohort descriptions using original patient data. Information about frequency of diagnosis (meningitis, meningoencephalitis, meningoencephalomyelitis, meningomyelitis), paresis, and fatality rates were extracted from full articles from selected abstracts. We included original articles published between 1975 and 2020, with cohort sizes of at least 20 TBE patients presumably infected by the European subtype of TBEV.

### 2.6. Ethical Statement

The study was approved by the local ethics committee of each center, and appropriate informed consent was obtained from all participating patients.

## 3. Results

### 3.1. Patient Characteristics and Findings in Acute Disease

A total of 1045 patients with TBE were recruited for genetic investigations (EU-TICK-BO); 430 of a single center were excluded as they only participated in the genetic analysis; 17 patients were excluded due to their date of admission before 2010 and 43 because of an incomplete basic data set after quality control. Finally, 555 (420 prospectively and 135 retrospectively recruited) cases were eligible for clinical description (Table 1), with ages ranging from 11 months to 88 years (median of 50 years, interquartile range (IQR) 36–61). Overall, 23 patients were younger than 15 years; 144 (25.9%) patients had comorbidities or conditions: 95 (17.1%) had a cardiovascular disease, 17 (3.1%) had a pre-existing neurological disease, 11 (1.9%) had a respiratory disease, 10 (1.8%) had a hematological disease, and 8 (1.4%) had other comorbidities (5 patients with renal disease, 2 patients with immunodeficiency and 1 patient with immunosuppression treatment after organ transplant). Three (0.5%) patients were pregnant.

Sixteen (2.9%) patients had TBE despite being vaccinated. Out of these, seven were vaccinated as recommended, six had no complete basic vaccination, and three had their last booster vaccination not according to the recommendation. Most patients had a biphasic course of disease (65.1%), with various symptoms or signs, including fever, fatigue, malaise, headache, body pain, pharyngitis, and gastrointestinal symptoms (Table 1).

**Figure 1:**
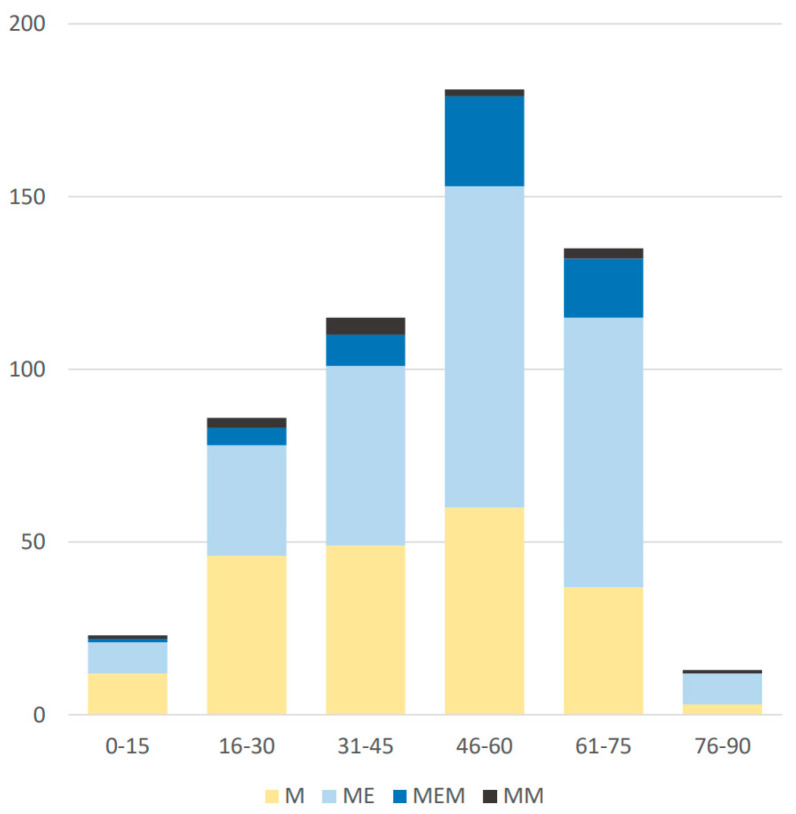
Distribution of clinical diagnosis according to age groups.

A clinical diagnosis was assigned to 553 of 555 patients; 207 (37.3%) had meningitis (M), 241 (43.4%) moderate meningoencephalitis (ME mod.), 32 (5.8%) severe meningoencephalitis (ME sev.), 15 (2.7%) meningomyelitis (MM), 46 (8.3%) meningoencephalomyelitis (MEM) with moderate encephalitis, and 13 (2.3%) meningoencephalomyelitis with severe encephalitis. In two patients, a differentiation of peripheral from central paresis was not possible, and these patients were classified as “unknown”. The distribution of diagnoses according to age is shown in Figure 1.

### 3.2. Patients with Pareses at Acute Phase of Disease

In total, 56 (10.1%) patients had pareses of extremities, of whom 41 (7.4%) had peripheral pareses of extremities, 13 (2.3%) central pareses of extremities, and 2 had unknown peripheral or central pareses of extremities (Table 2). A detailed distribution of pareses of extremities is shown in Table 2. Twenty-five (4.5%) patients had cranial nerve palsy. Nineteen of them had a single cranial nerve palsy, and six had multiple cranial nerve palsies. Further details about cranial nerve palsies were not recorded.

### 3.3. Spinal Involvement

Spinal involvement was observed, including paresis of extremities (as reported), disturbance of sensibility (*n* = 20), bladder dysfunction (*n* = 20), pain in extremities (*n* = 17), respiratory paresis (*n* = 9), and rectal dysfunction (*n* = 9).

### 3.4. Correlation of Clinical Diagnosis

The following characteristics and findings were significantly associated with clinical diagnosis: patients with M were younger than patients with ME (*p* < 0.0001), and patients with MEM (*p* = 0.002) had lower rates of comorbidities (OR = 0.52, *p* = 0.004 and OR = 0.46, *p* = 0.03, respectively) and were less often admitted to the intensive care unit (ICU) (OR = 0.07, *p* < 0.0001 and OR = 0.10, *p* = 0.006, respectively) (Table 3). At discharge, patients with M had higher rates of complete recovery than patients with ME (OR = 2.64, *p* = 0.0006) and patients with MEM (OR = 13, *p* < 0.0001). Further, patients with ME had higher rates of recovery compared to patients with MEM (OR = 2.6, *p* = 0.0006) (Table 3). Accordingly, patients with M had lower rates of incomplete recovery than patients with ME (OR = 0.38, *p* = 0.0006) and patients with MEM (OR = 0.07, *p* < 0.0001) (Table 3). Further patient characteristics, including gender, body mass index (BMI), and vaccination status, were not associated with specific clinical diagnoses.

### 3.5. Outcome at Discharge

We recorded the outcome at the discharge of 298 patients; 117 (39%) had a complete recovery, 176 (59%) an incomplete recovery, and 5 (2%) died. Based on the treating physician’s assessment, 96 (32%) patients were discharged with “complete recovery expected”, while 80 (27%) were discharged with “no recovery expected” (Table 4).

Symptoms and signs of 121 patients discharged with incomplete recovery were recorded (Table 5); 36 (30%) had mild subjective symptoms, 28 (23%) had severe subjective symptoms, and 57 (47%) had objective signs, of whom 25 (20.5%) had one objective sign, 25 (20.5%) had two objective signs, and 7 (6%) had three or more objective signs. Headache (93% of patients) was a predominant subjective symptom, followed by decreased concentration (47%). Objective signs included tremor (31%), ataxia (22%), and pareses of extremities (16%) (Table 5).

### 3.6. Correlation of Outcome at Discharge

While there was no significant difference in ICU admission between patients with complete and incomplete recovery (*p* = 0.227), deceased patients were admitted more often to the ICU than patients with compete recovery (OR = 49.9, *p* = 0.0002) and patients with incomplete recovery (OR = 30, *p* = 0.001). Further, deceased patients had higher rates of central paresis than patients with complete recovery (OR = infinity, *p* = 0.041). Patients with incomplete recovery were diagnosed less often with meningitis than patients with complete recovery (OR = 0.3, *p* < 0.0001) and more often with meningoencephalomyelitis (OR = 7, *p* < 0.0001), central paresis (OR = infinity, *p* = 0.012), peripheral paresis (OR = 19, *p* < 0.0001), cranial palsy (OR = 5.3, *p* = 0.02), and pain in extremities (OR = 12, *p* = 0.002). (Table 4).

### 3.7. Patients with Fatal Outcome

Patient 1 was a 39-year-old male with a kidney transplant and immunosuppression. He developed a severe diffuse CNS dysfunction (GCS = 3), needed mechanical ventilation, and died 4 weeks after the onset of TBE. The cause of death was described as extensive brain inflammatory damage and destabilization of brain functions. Patient 2 was a 47-year-old male with arterial hypertension and hyperuricemia. He had severe diffuse CNS dysfunction (GCS = 3) and died from elevated intracranial pressure 4 weeks after the onset of disease. Patient 3 was a 48-year-old female with arterial hypertension. She had severe diffuse CNS dysfunction (GCS = 3) and a central paresis. She died 4 weeks after the onset of TBE. The cause of death was described as extensive brain inflammatory damage and destabilization of brain functions. Patient 4 was a 66-year-old male with arterial hypertension. He had a disturbance of sensibility, a paresis of the left arm, and neck muscle dysfunction. He died within 4 weeks of TBE onset, caused by respiratory insufficiency. Patient 5 was a 79-year-old female with arterial hypertension who died after 4 weeks of TBE onset. The cause of death was not recorded.

### 3.8. Blood and CSF Findings

No differences in blood parameters were observed when patients were grouped according to diagnosis (Figure S1). In CSF, we observed significant differences in protein concentration (M lower than ME, *p* < 0.001; M lower than MEM, *p* < 0.001) and lactate concentration (M lower than MEM, *p* < 0.05; ME lower than MEM, *p* < 0.05). There were no significant differences in leukocyte, neutrophil, and lymphocyte counts and glucose concentration (Figure S2).

### 3.9. Literature Review

In total, 37 clinical studies published between 1975 and 2020 were included [3,4,6,7,10,11,16,17,18,19,20,21,22,23,24,25,26,27,28,29,30,31,32,33,34,35,36,37,38,39,40,41,42,43,44,45,46] (Table 6). The distribution of diagnoses showed the frequency of M ranging from 7 to 78%, ME from 13 to 84%, and MEM from 3 to 11%. In children, M ranged from 63 to 97%, ME from 1.5 to 37%, and MEM from 0 to 4%. Only one study reported on MM (Table S3). The description of paresis showed a frequency of overall paresis ranging from 1.6 to 10%, overall paresis of extremities ranging from 0.7 to 15.1%, peripheral paresis of extremities ranging from 0 to 10.6%, central paresis of extremities ranging from 0.9 to 2.9%, and cranial nerve palsies ranging from 1 to 11.3% (Table S4). In total, 34 clinical studies, with a total of 35,875 patients, reported on mortality rates ranging from 0 to 6.3%, with no significant improvement over the decades. Children had significantly lower fatality rates, ranging from 0 to 0.2% (Table S5).

## 4. Discussion

This is the first multicenter report on clinical findings of TBE patients from six highly endemic European countries. We confirm the high rates of patients with encephalitic disease caused by TBEV in European countries, as previously described by single-center studies or national studies [3,4,16,20,34]. The analysis of patient characteristics confirmed higher age as a risk factor for severe disease, as has been shown before [4,20,24]. Older age groups had a higher risk for meningoencephalitis and meningoencephalomyelitis. Further, pre-existing comorbidities were identified as risk factors for meningoencephalitis and meningoencephalomyelitis. Similar to previous reports, we observed a relatively high proportion of patients with moderate (43.4%) and severe meningoencephalitis (5.8%) [34,39,47]. Additionally, a high proportion of patients with myelitis (13.1%) was seen, whereas 2.7% had myelitis without signs of encephalitis. Interestingly, case reports about MM have been published, but the literature review showed only one clinical study describing MM as a separate clinical diagnosis, [18] suggesting a likely underreporting of patients with MM. A significant proportion of patients (10.1%) developed paresis of extremities, which was consistent with previous publications suggesting a rate from 0.7% to 15.1% [7,22]. Upper extremities were predominantly affected, as shown before [8]. Interestingly, a differentiation between peripheral and central paresis was rarely described, and paresis was often summarized as limb paresis. Cranial nerve palsies were seen in 25 (4.5%) patients, which was consistent with previous publications suggesting a rate of 1% to 11.3% [7,22]. A fatal course of disease was seen in 5 (1%) patients. Previous reports show similar death rates ranging from 0 to 1.44% (Table S5). In our study, patients with a fatal course of disease were aged between 39 to 79 years. Although we observed severe cases, none of the pediatric cases died. A relatively low mortality rate in children and adolescents has been reported before [31,44,45]. According to our findings and to the findings of the literature review, no significant decrease in mortality rates during the last decades was found. The analysis of routine laboratory blood parameters showed no significant differences. However, patients with M had significantly lower CSF protein and lactate concentrations compared to patients with ME or MEM. These findings are consistent with previous studies that reported elevated CSF protein levels in patients with ME [48]. Another study found an association of high CSF protein levels with elevated rates of sequelae [5]. CSF lactate levels are generally reported to be within a normal range [48,49], while publications on patients with MEM also describe elevated CSF lactate levels [50]. More than half of the patients were discharged with incomplete recovery (176/298 patients, 59%). This included a high proportion (80/298 patients, 27%) with no expectation of complete recovery at discharge according to the clinician’s assessment. Previous publications also describe high numbers of patients with sequelae after TBE infection [6,8,10,51,52]. Most impressively, TBE causes pareses of extremities in 10.1% of patients, with incomplete recovery of paresis at discharge. This impairment is a major factor for loss of function and loss of life quality and attributes to the high burden of TBE disease [53]. Follow-up studies showed persistence of pareses in more than 50% of patients one month after discharge [5,54]. Another follow-up study showed that only a few patients had a resolution of pareses within 2–7 years after discharge [52]. Further findings at discharge included a broad range of subjective symptoms (headache, decreased concentration, decreased stress tolerance) and objective signs (tremor and ataxia). A Slovenian study investigated the burden of TBE, which amounted to 3.1 disability-adjusted life years (DALYs) per TBE case [53]. In summary, TBE causes severe sequelae and quantifiable long-lasting limitations in daily life.

Our cohort included 16 patients with a history of previous TBE vaccination; 7 patients were fully vaccinated while 9 were incompletely vaccinated or missed receiving a booster vaccination according to recommendations. In contrast to previous studies, we observed a high proportion of children and adolescents with vaccination breakthrough infections [55,56]. In total, 9 of 16 vaccine breakthroughs were reported in patients younger than 20 years of age, and 7 of them were from a single center in Austria. This high proportion of children and adolescents with vaccination breakthrough infections might be the result of a special screening program of the Department of General Paediatrics, Medical University of Graz, to improve diagnostics in children with encephalitis and raise awareness for TBE breakthrough infections.

Literature about the severity of TBE breakthrough infection is inconsistent. Previous case reports and case series studies have described a more severe course of disease in patients with vaccination breakthrough infection, while a recent publication investigating a large cohort in Germany did not substantiate this finding [57,58]. The analysis of our cohort showed no significant difference in the distribution of diagnosis or in outcome at discharge when comparing vaccinated or unvaccinated patients; however, the number of breakthrough cases was low.

Although this analysis was carried out conscientiously, there may be potential limitations. According to viral epidemiological data, in all patients with TBE infections, the European TBEV subtype was presumed, though a differentiation of European, Siberian, and Far Eastern subtypes was not made due to non-feasibility in clinical routine. Possible coinfections with borrelia were excluded at each individual center according to clinical routine. Borrelia-specific diagnostic results were neither recorded nor reviewed by the lead study center. In this study, the true incidence of sequelae of our patients remains unknown. We only recorded the outcome at discharge since standardized follow-up investigations in all patients are of limited feasibility in routine patient care. Further investigation, including a detailed follow-up protocol, will be needed to study this subject. Further, the categorization of patients with incomplete outcomes at discharge for expected recovery is subjective to the corresponding investigator and is influenced by personal experience.

## 5. Conclusions

This is the first international multicenter study of patients with TBE from different European countries. We observed high rates of patients with encephalitis and high rates of patients with lasting signs and symptoms at discharge. The comparison with previously published cohorts showed a likely underreporting of patients with meningomyelitis and patients with central paresis, which might be caused by different case record forms. Therefore, we suggest the use of a uniform case record form to monitor the full spectrum of disease and to raise awareness for disease prevention, particularly in countries with low vaccination rates.

## Figures and Tables

**Table 1:** Details of TBE patients, including basic characteristics, course of disease, severity of acute disease, paresis and spinal involvement, and outcome at discharge.

Basic Characteristics	All Patients (*n* = 555)
gender, female (%)	223 (40)
age, years (range)	50 (0–88)
BMI in adults, median (IQR)	26 (23–29) *
comorbidity (%)	144 (26)
TBE vaccination–any (%)	16 (2.9) *
tick bite noticed (%)	273 (61) *
transmission by dairy products (%)	11 (2)
**course of disease**	
biphasic course (%)	329 (65) *
hospital stay (days), median (IQR)	10 (8–13) *
ICU admission (%)	38 (7) *
ICU length of stay median, days (IQR)	5 (2–8) *
**diagnosis**	
meningitis (%)	207 (37.3)
meningoencephalitis, moderate (%)	241 (43.4)
meningoencephalitis, severe (%)	32 (5.8)
meningomyelitis (%)	15 (2.7)
meningoencephalomyelitis, moderate encephalitis (%)	46 (8.3)
meningoencephalomyelitis, severe encephalitis (%)	13 (2.3)
unknown (%)	2 (0.2)
**neurological deficiencies**	
peripheral paresis (%)	41 (7.4)
central paresis (%)	13 (2.3)
unknown peripheral or central paresis (%)	2 (0.2)
single cranial palsy (%)	19 (3.4)
multiple cranial palsies (%)	6 (1.1)
disturbance of sensibility (%)	20 (5) *
bladder dysfunction (%)	20 (5) *
pain in extremities (%)	17 (4) *
respiratory paresis (%)	9 (2) *
rectal dysfunction (%)	9 (2) *
**outcome at hospital discharge**	
complete recovery (%)	117 (39) *
incomplete recovery (%)	176 (59) *
death (%)	5 (2)

* Number of available observations: all patients: *n* = 555, except BMI (*n* = 315), TBE vaccination status (*n* = 546), tick bite noticed (*n* = 444), course of illness (*n* = 504), length of hospital stay (*n* = 316), ICU admission (*n* = 554), length of ICU stay (*n* = 34), respiratory paresis (*n* = 419), bladder dysfunction (*n* = 420), rectal dysfunction (*n* = 419), disturbance of sensibility (*n* = 418), pain in extremities (*n* = 414), and outcome at hospital discharge (*n* = 298).

**Table 2:** Findings in patients with TBE in the initial phase and in the neurological phase.

Initial Phase	*n* (%)
no initial phase	175 (35)
fever	275 (55)
fatigue	221 (44)
malaise	188 (37)
headache	211 (42)
body pain	134 (27)
pharyngitis	70 (14)
gastrointestinal symptoms	58 (12)
**Neurological Phase**	
fever	501 (94)
headache	509 (95)
nausea or vomiting	323 (61)
nuchal rigidity	396 (74)
positive Kernig sign	208 (40)
disturbance of consciousness	134 (24)
ataxia	206 (37)
tremor	210 (38)
single cranial nerve palsy	20 (4)
dysphagia	9 (2)
severe CNS dysfunction (GCS < 9)	13 (2)
seizures	8 (1)
central paresis	10 (2)
respiratory failure (in need of mechanical ventilation)	7 (1)
multiple cranial nerve palsies	6 (1)
bulbar palsy	15 (3)

CNS = central nervous system; GCS = Glasgow Coma Score. Number of available observations: in initial phase: initial phase recorded (*n* = 505), including fever (*n* = 328), fatigue (*n* = 326), malaise (*n* = 327), headache (*n* = 329), body pain (*n* = 327), pharyngitis (*n* = 326), gastrointestinal symptoms (327); findings in neurological phase: fever (*n* = 535), headache (*n* = 534), nausea or vomiting (*n* = 530), nucheal rigidity (*n* = 532), positive Kernig’s sign (*n* = 521), disturbance of consciousness, ataxia, tremor, single cranial nerve palsy and dysphagia (*n* = 553), severe CNS dysfunction (*n* = 552), seizure (*n* = 554), central paresis (*n* = 553), respiratory failure (*n* = 553), multiple cranial nerve palsies (*n* = 552), and bulbar palsy (*n* = 552).

**Table 3:** Comparison of patient characteristics, course of disease, and result at discharge from hospital according to clinical diagnosis.

	Meningitis (*n* = 207)	Meningoencephalitis (*n* = 273)	Meningomyelitis (*n* = 15)	Meningoencephalo-myelitis (*n* = 58)	Significance
**patient characteristics**					
gender female (%)	77 (37)	124 (45)	4 (27)	18 (31)	*p* = 0.074
age median (IQR)	44 (29–57)	53 (41–63)	45 (30–64)	54 (44–63)	***p*< 0.0001** *
BMI in adults, median (IQR)	25 (23–29)	26 (23–28)	24 (22–27)	26 (24–30)	*p* = 0.52
comorbidity (%)	38 (18)	82 (30)	5 (33)	19 (33)	*p* = 0.011 *
TBE vaccination, any (yes/no)	4 / 203	9/260	1 / 13	2 / 55	*p* = 0.40
tick bite noticed (yes/no)	98 / 55	138/95	9 / 1	29 / 20	*p* = 0.21
transmission by dairy products (%)	6 (3)	4 (1)	0	1 (2)	n.a.
**course of disease**					
biphasic course (yes/no)	116 / 69	168 / 81	9 / 5	36 / 20	*p* = 0.77
hospital stay median (IQR)	9 (8–11), *n* = 85	10 (8–13), *n* = 187	12 (10–18), *n* = 6	11 (9–17), *n* = 38	***p*= 0.013** *
ICU admission (%)	2 (1)	30 (11)	1 (7)	5 (9)	***p*< 0.0001** *
ICU length of stay median, days (IQR)	3	5 (2–7)	n.a.	4 (2–12)	*p* = 0.35
**outcome at discharge from hospital**					
complete recovery (%)	50 (60)	60 (36)	3 (43)	4 (10)	***p*< 0.0001** *
incomplete recovery (%)	33 (40)	104 (62)	4 (57)	35 (88)	***p*< 0.0001** *
death (%)	0	4 (2)	0	1 (3)	*p* = 0.28

Abbreviations: n.a., not applicable. * Details on significance: age median: M vs. ME (*p* < 0.0001) and M vs. MEM (*p* = 0.002); comorbidity: M vs ME (OR = 0.52, CI = 0.32–0.82; *p* = 0.004); M vs. MEM (OR = 0.46, CI = 0.23–0.94; *p* = 0.03,); hospital stay: M vs. MEM (*p* = 0.023); ICU admission: M vs. ME (OR = 0.07, CI = 0.009–0.31; *p* < 0.0001) and M vs. MEM (OR = 0.10, CI = 0.01–0.7; *p* = 0.006); complete recovery at hospital discharge: M vs. ME (OR = 2.64, CI = 1.5–4.7; *p* = 0.0006); M vs. MEM (OR = 13, CI = 4.1–55; *p* < 0.0001); ME vs. MEM (OR = 2.6, CI = 1.5–4.7; *p* = 0.0006); incomplete recovery at hospital discharge: M vs. ME (OR = 0.38, CI = 0.21–0.68; *p* = 0.0006,); M vs. MEM (OR = 0.07, CI = 0.02–0.24; *p* < 0.0001).

**Table 4:** Comparison of patients with complete recovery, incomplete recovery, and death.

Basic Characteristics	Complete Recovery (*n* = 117)	Incomplete Recovery (*n* = 176)	Death (*n* = 5)	Significance
female (%)	48 (41)	80 (45)	2 (40)	*p* = 0.74
median age in years (IQR)	50 (28–64)	51 (40–61)	48 (43–73)	*p* = 0.46
comorbidity (%)	36 (31)	54 (31)	4 (80)	*p* = 0.076
TBE vaccination (%)	8 (7)	7 (4)	0	*p* = 0.45
tick bite noticed (%)	77 (66)	103 (59)	3 (60)	*p* = 0.42
diary product (%)	2 (2)	5 (3)	0	*p* = 0.73
**course of Disease**				
biphasic course	81 (70)	114 (65)	3 (60)	*p* = 0.63
median BMI (IQR)	26 (23–29)	26 (24–29)	28 (22–35)	*p* = 0.95
median hospital stay in days (IQR)	10 (8–12)	10 (8–14)	10 (9–11)	*p* = 0.12
ICU admission (%)	8 (7)	20 (11)	4 (80)	*p* = 0.0003 *
median days on ICU (IQR)	5 (3–5)	4 (2–7)	10 (9–11)	*p* = 0.18
**diagnosis**				
meningitis (%)	50 (42)	33 (19)	0	*p* < 0.0001 *
meningoencephalitis (%)	60 (51)	104 (59)	4 (80)	*p* = 0.26
meningomyelitis (%)	3 (3)	4 (2)	0	*p* > 0.99
meningoencephalomyelitis (%)	4 (4)	35 (20)	1 (20)	*p* < 0.0001 *
**pareses**				
central paresis (%)	0	9 (5)	1 (20)	*p* = 0.0036 *
peripheral paresis (%)	1 (2)	25 (14)	1 (20)	*p* < 0.0001 *
cranial nerve palsy (%)	2 (2)	15 (9)	0	*p* = 0.049 *
**spinal involvement**				
respiratory paresis (%)	1 (1)	7 (4)	0	*p* = 0.26
bladder dysfunction (%)	4 (3)	13 (7)	0	*p* = 0.40
rectal dysfunction (%)	1 (1)	6 (3)	0	*p* = 0.33
disturbance of sensibility	1 (1)	6 (3)	1 (20)	*p* = 0.06
pain in extremities	1 (1)	16 (9)	0	*p* = 0.006 *

* Details about significance: ICU admission: death vs complete rec. (OR = 49.9, CI = 4.3–2651; *p* = 0.0002); death vs incompl. rec. (OR = 30, CI = 2.8–1530; *p* = 0.001); diagnosis meningitis: incomplete rec. vs complete rec. (OR = 0.3, CI = 0.17–0.54; *p* < 0.0001); diagnosis meningoencephalomyelitis: incomplete rec. vs complete rec (OR= 7, CI = 2.4–28; *p* < 0.0001); central paresis: incomplete rec. vs complete rec (OR = infinity, CI = 1.4-infinity; *p* = 0.012); death vs. complete rec. (OR = infinity, CI= 0.6-infinity, *p* = 0.041); peripheral paresis: incomplete rec. vs complete rec. (OR= 19, CI = 3–792; *p* < 0.0001); cranial nerve palsy: incomplete rec. vs complete rec. (OR = 5.3, CI = 1.2–49; *p* = 0.02); pain in extremities: incomplete rec. vs complete rec. (OR = 12, CI = 1.7–490, *p* = 0.002).

**Table 5:** Symptoms and signs of TBE in patients discharged with incomplete recovery.

	Patients (*n* = 121)
Subjective Symptoms	*n* (%)
headache	112 (93)
decreased concentration	57 (47)
decreased stress tolerance	27 (22)
increased irritability	21 (17)
decreased memory	32 (26)
emotional instability	24 (20)
sleep disturbance	39 (32)
**objective Signs**	
dysarthria	2 (2)
dysphagia	0
diplopia	2 (2)
hemiparesis	2 (2)
cranial nerve palsy—ocular	0
cranial nerve palsy—facial	3 (2)
cranial nerve palsy—pharyngeal	0
ataxia	27 (22)
tremor	38 (31)
hemihypaesthesia	2 (2)
paresis of extremities	19 (16)
disturbance of sensibility	4 (3)
bladder dysfunction	1 (1)
bowel dysfunction	0
sexual dysfunction	0

**Table 6:** Summary of results from the literature review and our study.

	Literature Review, Range (%)	EU-TICK-BO Cohort (%)
**diagnoses**		
meningitis	7–78	37
meningoencephalitis	13–84	49
meningoencephalomyelitis	3–11	11
meningomyelitis	0	3
**pareses**		
… of extremities, all	0.7–15.1	10.1
… of extremities, peripheral	0–10.6	7.4
… of extremities, central	0.9–2.9	2.3
… cranial nerve palsies	1–11.3	4.5
**mortality rate**	0–6.3	0.9

## Data Availability

The data that supports the findings of this study are available from the corresponding author upon reasonable request.

## Supplementary Materials

The following are available online at https://www.mdpi.com/article/10.3390/microorganisms9071420/s1, Suppl. Case Record Form (CRF), Suppl. Table S1. Patient recruitment sites, Suppl. Table S2. Distribution of paresis in patients with peripheral and central paresis of extremities, Suppl. Table S3. TBE patients assigned to M, ME, MEM, MM or other diagnosis according to 20 clinical studies from European countries published during 1975–2019., Suppl. Table S4. TBE patients with paresis according to 23 clinical studies from European countries published during 1975–2019., Suppl. Table S5. TBE Fatality rate according to clinical studies from 34 European countries published during 1975–2020., Suppl. Figure S1. Findings in blood on admission., Suppl. Figure S2. Findings in cerebrospinal fluid (CSF). Tukey plot with whiskers. * indicates *p* ≤ 0.05, ** indicates *p* ≤ 0.005 and *** indicates *p* ≤ 0.0005.
