# Supplementary material for: Clinical Characteristics of Patients with Tick-Borne Encephalitis (TBE): A European Multicentre Study from 2010 to 2017
Source: Microorganisms. 2021 Jun 30;9(7):1420. doi: 10.3390/microorganisms9071420 (PMC8306415; doi:10.3390/microorganisms9071420)
Supplement: Supplementary file 1 [file microorganisms-09-01420-s001.zip › microorganisms-1275154-supplementary.pdf]

Supplement file of "Clinical characteristics of patients with Tick-borne encephalitis (TBE): A European multicentre study from 2010 to 2017"

Suppl. Case Record Form (CRF)

1. **Patient Details**
   1.1. Patient ID
   1.2. Consent signed (Y/N)
   1.3. Gender (male/female/other)
   1.4. Date of birth (day/month/year)
   1.5. Weight (kg)
   1.6. Height (cm)
   1.7. Ethnicity
      1.7.1. North/Mid/East European
      1.7.2. South European (Mediterranean countries and Portugal)
      1.7.3. Roma
      1.7.4. African
      1.7.5. Asian
      1.7.6. Middle Eastern
      1.7.7. Meso/South American
      1.7.8. Others
   1.8. Admitted to study centre (day/month/year)
   1.9. Admitted to ICU (Y/N)
      1.9.1. If yes, date (day/month/year)

2. **Medical history**
   2.1. Parents consanguineous (Y/N)
   2.2. Familial history
      2.2.1. Further TBE cases in family (Y/N)
         2.2.1.1. Affected persons
            2.2.1.1.1. Father (Y/N)
            2.2.1.1.2. Mother (Y/N)
            2.2.1.1.3. Siblings (specify number)
            2.2.1.1.4. Grandparents (specify number)
            2.2.1.1.5. Other family members (specify)

   2.3. Past medical history
      2.3.1. Immune problems
         2.3.1.1. Immune deficiency (specify)
         2.3.1.2. Haemato-/ Oncologic disease (specify)
         2.3.1.3. Immunosuppressive agents (specify)
         2.3.1.4. Organ transplantation (specify)
      2.3.2. Chronic conditions
         2.3.2.1. Cardiovascular disease (specify)
         2.3.2.2. Neurological disease (specify)
         2.3.2.3. Respiratory disease (specify)
         2.3.2.4. Kidney disease (specify)
      2.3.3. Pregnancy during TBE
      2.3.4. Other relevant details (please specify)

   2.4. Virus transmission
      2.4.1. Tick bite within four weeks after onset of disease (Y/N)

2.4.2. Transmission of TBE via unpasteurized dairy products (Y/N)
2.4.3. Possible Exposure: stay in endemic area (Y/N)

2.5. Immunisations

2.5.1. TBE immunisation (Y/N)
2.5.2. Vaccinated according to recommendations (Y/N)
2.5.3. Total numbers of immunisations against TBE (specify)
2.5.3.1. Number of vaccination with Encepur (specify)
2.5.3.2. Numbers of vaccination with FSME Immun (specify)
2.5.3.3. Interval between last TBE vaccination and onset of disease
2.5.3.3.1. < 1 year
2.5.3.3.2. 1-2 years
2.5.3.3.3. 3-5 years
2.5.3.3.4. 6-9 years
2.5.3.3.5. >9 years
2.5.4. Vaccine failure (Y/N)
2.5.5. Immunisations against Yellow fever or Japan B Encephalitis (Y/N)

3. TBE diagnostic criteria

3.1. Detection of TBE-specific IgM and IgG antibodies in serum* (Y/N)
3.1.1. Date (day/month/year)
3.2. Detection of TBE-specific IgM in CSF (Y/N)
3.3. Detection of TBE-specific IgG in CSF (Y/N)
3.4. Sero-conversion or significant increase of TBE-specific antibodies in paired serum samples* (Y/N)
3.5. Isolation of TBE virus from clinical specimen (Y/N)
3.6. Detection of TBE viral nucleic acid in clinical specimen (Y/N)
3.7. Confirmation of diagnosis by neutralization assay (Y/N)

** Remark for any serological testing: interpretation of serological results has to be according to the vaccination status and previous exposure to other flaviviral infections*

4. Initial Phase

4.1. Initial phase of TBE (Y/N)
4.2. Duration of initial phase (days)
4.3. Interval between tick bite and first symptoms (days)
4.4. Interval between tick bite and onset of neurologic symptoms (days)
4.5. Onset of neurological symptoms (day/month/year)

4.6. Symptoms of prodromal stage
4.6.1. Fever (Y/N)
4.6.2. Fatigue (Y/N)
4.6.3. Malaise (Y/N)
4.6.4. Headache (Y/N)
4.6.5. Body pain such as aching back and limbs (Y/N)
4.6.6. Pharyngitis (Y/N)
4.6.7. Gastrointestinal symptoms (Y/N)

5. Clinical Assessment

5.1. How many times was the patient classified by the investigator? (1/2/3 or more)

5.2. Glascow coma scale (GCS)

- 5.2.1. Eye opening response
  - 5.2.1.1. 4 points: spontaneous – open with blinking at baseline
  - 5.2.1.2. 3 points: to verbal stimuli, command, speech
  - 5.2.1.3. 2 points: to pain only (not applied to face)
  - 5.2.1.4. 1 point: no response
- 5.2.2. Motor response
  - 5.2.2.1. 6 points: obeys commands for movement
  - 5.2.2.2. 5 points: purposeful movement to painful stimulus
  - 5.2.2.3. 4 points: withdraw in response to pain
  - 5.2.2.4. 3 points: flexion in response to pain (decorticate posturing)
  - 5.2.2.5. 2 points: extension response in response to pain (decerebrate posturing)
  - 5.2.2.6. 1 point: no response
- 5.2.3. Verbal response
  - 5.2.3.1. 5 points: orientated
  - 5.2.3.2. 4 points: confused conversation, but able to answer question
  - 5.2.3.3. 3 points: inappropriate words
  - 5.2.3.4. 2 points: incomprehensible speech
  - 5.2.3.5. 1 point: no response

5.3. Maximum Value of Severity

- **5.3.1. Findings indicating meningitis**
  - 5.3.1.1. Lumbar puncture results indicate meningitis (Y/N)
  - 5.3.1.2. Body temperature >38°C (Y/N)
  - 5.3.1.3. Headache (Y/N)
  - 5.3.1.4. Nausea and/or vomiting (Y/N)
  - 5.3.1.5. Nuchal rigidity (Y/N)
  - 5.3.1.6. Positive Kernig sign (Y/N)
  - 5.3.1.7. Other (specify)
- 5.3.2. **Findings indicating moderate encephalitis**
  - 5.3.2.1. Ataxia (Y/N)
  - 5.3.2.2. Tremor (Y/N)
  - 5.3.2.3. Single cranial nerve paralysis (Y/N)
  - 5.3.2.4. Dysphagia (Y/N)
  - 5.3.2.5. Other (specify)
- **5.3.3. Findings indicating severe encephalitis**
  - 5.3.3.1. GCS<9 (Y/N)
  - 5.3.3.2. Seizures (Y/N)
  - 5.3.3.3. Central paresis (Y/N)
  - 5.3.3.4. Mechanical ventilation (Y/N)
  - 5.3.3.5. Multiple cranial nerve paralyses (Y/N)
  - 5.3.3.6. Number of effected extremities (1/2/3/4/unknown)
  - 5.3.3.7. Bulbar symptoms (Y/N)
- **5.3.4. Spinal nerve involvement**
  - 5.3.4.1. Paresis grade (5 is normal, 0 is complete paresis)
    - 5.3.4.1.1. Right arm (specify grade)
    - 5.3.4.1.2. Left arm (specify grade)
    - 5.3.4.1.3. Right leg (specify grade)

5.3.4.1.4. Left leg (specify grade)
5.3.4.2. Disturbance of sensibility (number of effected extremities)
5.3.4.3. Pain in extremities (number of effected extremities)
5.3.4.4. Respiratory muscle paresis (Y/N)
5.3.4.5. Bladder dysfunction (Y/N)
5.3.4.6. Rectal dysfunction (Y/N)
5.3.4.7. Other (specify)

5.3.5. Death

5.3.5.1. Within 4 weeks from onset of TBE infection (Y/N)
5.3.5.2. Beyond 4 weeks from onset of TBE infection (Y/N)
5.3.5.3. Due to elevated intracranial pressure (Y/N)
5.3.5.4. Due to respiratory insufficiency (Y/N)
5.3.5.5. Other (specify)

5.3.6. Diagnosis
5.3.6.1. Meningitis
5.3.6.2. Meningoencephalitis
5.3.6.3. Meningomyelitis
5.3.6.4. Meningoencephalomyelitis
5.3.6.5. Other (specify)

6. Radiological investigations

6.1. CT
6.1.1. Done (Y/N)
6.1.2. Date (day/month/year)
6.1.3. Lesions (Y/N)
6.1.4. Lesions in thalamus (Y/N)
6.1.5. Lesions in cerebellum (Y/N)
6.1.6. Lesions in striatum (Y/N)
6.1.7. Lesions in nuclei caudati (Y/N)
6.1.8. Other location of lesions or other finding (specify)

6.2. Brain MRI
6.2.1. Done (Y/N)
6.2.2. Date (day/month/year)
6.2.3. MRI technical details
6.2.3.1. Flair (Y/N)
6.2.3.2. T2 (Y/N)
6.2.3.3. T1 (Y/N)
6.2.3.4. Gadolinium (Y/N)
6.2.4. Lesions (Y/N)
6.2.4.1. Please specify
6.2.5. Localisation
6.2.5.1. Cortex (Y/N)
6.2.5.2. White matter (Y/N)
6.2.5.3. Basal ganglia (Y/N)
6.2.5.4. Thalamus (Y/N)
6.2.5.5. Brainstem (Y/N)
6.2.5.6. Cerebellum (Y/N)

6.2.6. Leptomeningeal enhancement (Y/N)
6.2.7. Mass effect (Y/N)
6.2.8. Number of lesions
6.2.9. Maximum diameter of largest lesion (mm)

6.3. Spinal MRI
6.3.1. Done (Y/N)
6.3.2. Date (day/month/year)
6.3.3. Description of findings
6.3.4. Lesions (Y/N)
6.3.4.1. Cervical
6.3.4.1.1. Number of lesion (specify)
6.3.4.1.2. Gadolinium enhancement (Y/N)
6.3.4.1.3. Mass effect (Y/N)
6.3.4.1.4. Maximum length (mm)
6.3.4.1.5. Maximum diameter (mm)
6.3.4.2. Thoracic
6.3.4.2.1. Number of lesion (specify)
6.3.4.2.2. Gadolinium enhancement (Y/N)
6.3.4.2.3. Mass effect (Y/N)
6.3.4.2.4. Maximum length (mm)
6.3.4.2.5. Maximum diameter (mm)
6.3.4.3. Leptomeningeal enhancement (Y/N)
6.3.4.4. Radicular enhancement (Y/N)
6.3.4.5. Spinal cord atrophy (Y/N)

7. Laboratory data
7.1. Blood (*to be collected at hospital admission / 24-48hr / recovery-convalescence*)
7.1.1. White cell count (G/L)
7.1.2. Neutrophils (G/L)
7.1.3. Lymphocytes (G/L)
7.1.4. Hemoglobin (g/dL)
7.1.5. Platelets (G/L)
7.1.6. C reactive protein (mg/L)
7.1.7. Glucose (mg/L)
7.1.8. ALT (U/L)
7.1.9. AST (U/L)
7.2. Cerebrospinal fluid (CSF)
7.2.1. CSF leukocytes count (/µL)
7.2.2. CFS neutrophils (/µL)
7.2.3. CSF lymphocytes (/µL)
7.2.4. CSF protein (mg/dL)
7.2.5. CSF glucose (mg/dL)
7.2.6. CSF lactate (mmol/L)

8. Outcome at hospital discharge

- 8.1. Date of discharge (day/month/year)
- 8.2. Full recovery (Y/N)
- 8.3. Incomplete recovery (Y/N)
  - 8.3.1. Recovery expected (Y/N)

8.4. New or increased symptoms/signs in comparison to situation prior to acute TBE

- 8.4.1. <u>Subjective symptoms</u>
  - 8.4.1.1. Headache (Y/N)
  - 8.4.1.2. Decreased concentration (Y/N)
  - 8.4.1.3. Decreased stress tolerance (Y/N)
  - 8.4.1.4. Increased irritability (Y/N)
  - 8.4.1.5. Decreased memory (Y/N)
  - 8.4.1.6. Emotional instability (Y/N)
  - 8.4.1.7. Sleep disturbance (Y/N)
- 8.4.2. <u>Objective symptoms</u>
  - 8.4.2.1. Dysarthria (Y/N)
  - 8.4.2.2. Dysphagia (Y/N)
  - 8.4.2.3. Diplopia (Y/N)
  - 8.4.2.4. Hemiparesis (Y/N)
  - 8.4.2.5. Cranial nerve palsy
    - 8.4.2.5.1. Ocular (Y/N)
    - 8.4.2.5.2. Facial (Y/N)
    - 8.4.2.5.3. Pharyngeal (Y/N)
  - 8.4.2.6. Ataxia (Y/N)
  - 8.4.2.7. Tremor (Y/N)
  - 8.4.2.8. Hemihypaesthesia (Y/N)
  - 8.4.2.9. Paresis of extremities (number of effected extremities)
  - 8.4.2.10. Disturbance of extremities (number of effected extremities)
  - 8.4.2.11. Bowel dysfunction (Y/N)
  - 8.4.2.12. Sexual dysfunction (Y/N)
  - 8.4.2.13. Bladder dysfunction (Y/N)
  - 8.4.2.14. Rectal dysfunction (Y/N)

Suppl. Table S1. Patient recruitment sites (alphabetical order)

Austria, Graz, Department of Neurology and Department of General Paediatrics (Medical University of Graz)

Czech Republic, Brno, Department of Infectious Diseases (University Hospital Brno) and Department of Children's Infectious Disease (Masaryk University)

Czech Republic, Ceske Budejovice, Department of Infectious Diseases (Hospital Ceske Budejovice)

Czech Republic, Plzen, Department of Infectious Disease and Travel Medicine (University Hospital Plzen)

Latvia, Riga, Neurology and Neurosurgery Department, Riga East University Hospital; Clinic and Department of infectious diseases, Children clinical university hospital (Riga Stradins University)

Lithuania, Kaunas, Department of Infectious Diseases (Lithuanian University of Health Sciences)

Poland, Bialystock, Department of Infectious Diseases and Neuroinfections (Medical University Bialystok)

Slovenia, Maribor, Department of Infectious Diseases and Febrile Conditions, (University Clinical Centre Maribor)

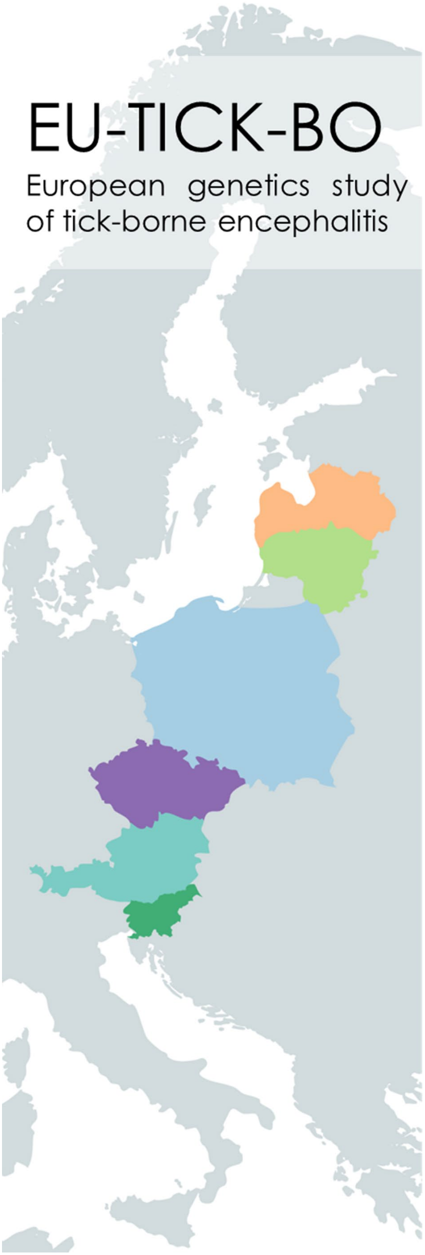

Suppl. Table S2. Distribution of paresis in patients with peripheral and central paresis of extremities

**location**	**number of patients**
peripheral paresis (n=41)	
arm, right	11
arm, left	13
arms, both	5
leg, right	0
leg, left	4
legs, both	5
arm left and leg left	1
all extremities	2
central paresis (n=13)	
hemiparesis, right	3
hemiparesis, left	2
hemiparesis, unkown side	1
arm, right	1
all extremities	1
unknown	5
unknown peripheral or central paresis (n=2)	
arms, both	1
legs, both and arm, right	1

Suppl. Table S3. TBE patients assigned to M, ME, MEM, MM or other diagnosis according to 20 clinical studies from European countries published during 1975-2019.

author	year published	country	reported period	total number of patients assessed	M (%)	ME (%)	MEM (%)	MM (%)	others (%)
Duniewicz [1]	1975	Czech Republic	1969-1972	589	40 (7)	492 (84)	57 (9)	0	0
Ackermann [2]	1979	Germany	1964-1977	51	23 (45)	19 (37)	1 (2)	8 (16)	0
Kaiser [3]	1996	Germany	1994-1995	300	150 (50)	116 (39)	21 (7)	0	13 (4) encephaloradiculitis
Kaiser [4]	1999	Germany	1994-1998	656	320 (49)	270 (41)	66 (10)	0	0
Kaiser [5]	2002	Germany	1991-2000	849	400 (47)	356 (42)	93 (11)	0	0
Grygrorczuk [6]	2002	Poland	1997-2001	152	51 (33.5)	89 (58.5)	12 (8)	0	0
Zimmermann [7]	2005	Swiss	1999-2004	572	140 (24)	284 (50)	35 (6)	0	81 (14) non-CNS, 32 (6) unsure neurological
Hansmann [8]	2006	France	1968-2003	61	33 (54)	21 (34)	0	0	2 (3) encephalitis, 2 (3) meningoradiculitis, 3 (5) abortive forms
Logar [9]	2006	Slovenia	2000-2004	448	163 (36)	269 (60)	16 (4)	0	0
Karelis [10]	2008	Lativa	1994-2006	236	183 (78)	31 (13)	22 (9)	0	0
Czupryna [11]	2011	Poland	1993-2008	687	282 (41)	353 (51)	52 (8)	0	0
Radzisauskiene [12]	2018	Lithuania	2005-2014	684	66 (10)	556 (81)	22 (3)	0	40 (6) encephalitis
Bogovic [13]	2018	Slovenia	2007-2012	717	231 (32)	445 (62)	41 (6)	0	0
Hellenbrand [14]	2019	Germany	2015-2018	1636	n.a. (31.5)	n.a. (17.5)	61 (4)	0	770 (47) non-CNS
Krawczuk [15]	2020	Poland	2004–2015	601	305 (51)	246 (41)	50 (8)	0	0
Children only									
Lesnicar [16]	2003	Slovenia	1959-2000	371	232 (63)	139 (37)	0	0	0
Fritsch [17]	2008	Austria	1981-2005	116	92 (79)	24 (21)	0	0	0
Stähelin-Massik [18]	2008	Swiss	2000-2004	55	37 (67)	16 (29)	2 (4)	0	0
Krbkova [19]	2015	Czech	1993-2012	153	133 (77)	22 (13)	0	0	1 (1) encephalitis
Krawczuk [15]	2020	Poland	2004–2015	68	66 (97)	1 (1.5)	1 (1.5)	0	0

Suppl. Table S4. TBE patients with paresis according to 23 clinical studies from European countries published during 1975-2019.

author	year published	country	reported period	total number of patients assessed	paresis: overall (%)	extremities: overall (%)	extremities: peripheral (%)	extremities: central (%)	cranial nerve paresis (%)
Duniewicz [1]	1975	Czech Republic	1969-1972	589	10 (1.6)	n.a.	n.a.	n.a.	n.a.
Wahlberg [20]	1989	Island and Finland	1959-1987	126	8 (6.3)	4 (3.2)	n.a.	n.a.	4 (3.2)
Holmgren [21]	1990	Sweden	1956-1989	1116	n.a. (10)	n.a.	n.a.	n.a.	n.a.
Kaiser [3]	1996	Germany	1994-1995	300	n.a.	n.a.	34 (11.3)	8* (2.7)	4 (1.3)
Günther [22]	1997	Sweden	1991-1993	85	n.a.	n.a.	9 (10.6)	0	2 (2.4)
Kaiser [4]	1999	Germany	1994-1998	656	n.a.	99 (15.1)	n.a.	n.a.	74 (11.3)
Mickiene [23]	2002	Lithuania	1998-1999	133	n.a.	n.a.	5 (3.8)	3 (2.3)	7 (5.3)
Grygorczuk [6]	2002	Poland	1997-2001	152	16 (10)	11 (7)	n.a.	n.a.	5 (3)
Zimmermann [7]	2005	Swiss	1999-2004	584	10 (1.7)	4 (0.7)	n.a.	n.a.	6 (1.0)
Hansmann [8]	2006	France	1968-2003	64	n.a.	2 (3.1)	n.a.	n.a.	5 (7.8)
Logar [9]	2006	Slovenia	2000-2004	448	17 (3.8)	16 (3.6)	n.a.	n.a.	5 (1.1)
Hansson [24]	2011	Sweden	2003-2008	32	n.a.	n.a.	n.a.	n.a.	1 (3.3)
Wahlberg [25]	2006	Finland (Åland)	1959-2005	301	13 (4.3)	n.a.	n.a.	n.a.	n.a.
Karelis [10]	2008	Latvia	1994-2006	100	n.a.	n.a.	n.a.	n.a.	11 (11)
Czupryna [11]	2011	Poland	1993-2008	621	53 (8.5)	40 (6.4)	n.a.	n.a.	19 (3.1)
Rezza [26]	2015	Italy	2000-2013	367	19 (5.2)	n.a.	n.a.	n.a.	n.a.
Radzisauskiene [12]	2018	Lithuania	2005-2014	712	n.a.	45 (6.3)	24 (3.4)	21 (2.9)	75 (10.5)
Krawczuk [15]	2020	Poland	2004–2015	601	n.a.	79 (13)	n.a.	n.a.	60 (10)
Children only									
Lesnicar [16]	2003	Slovenia	1959-2000	371	n.a.	11 (2.9)	n.a.	n.a.	14 (3.8)
Fritsch [17]	2008	Austria	1981-2005	116	n.a.	1 (0.9)	0	1 (0.9)	2 (1.7)
Krbkova [19]	2015	Czech	1993-2012	170	n.a.	n.a.	n.a.	n.a.	6 (3.5)
Stähelin-Massik [18]	2008	Swiss	2000-2004	55	n.a.	n.a.	n.a.	n.a.	n.a.
Krawczuk [15]	2020	Poland	2004–2015	68	n.a.	1 (1.5)	n.a.	n.a.	1 (1.5)

**reported as hemiparesis*

Suppl. Table S5. TBE Fatality rate according to clinical studies from 34 European countries published during 1975-2020.

author	year published	country	reported period	patients investigated	fatality rate (%)
Duniewicz [1]	1975	Czech Republic	1969-1972	633	5 (0.8)
Ackermann [2]	1979	Germany	1964-1977	51	2 (3.9)
Wahlberg [20]	1989	Island and Finland	1959–1987	108	0
Holmgren [21]	1990	Sweden	1956-1989	1 116	5 (0.4)
Köck [27]	1992	Austria	1987-1990	117	0
Haglund [28]	1996	Sweden	1978/1987	143	2 (1.4)
Kaiser [3]	1996	Germany	1994-1995	300	4 (1.3)
Kaiser [29]	1997	Germany	1990-1995	63	4 (6.3)
Günther [22]	1997	Sweden	1991-1993	85	0
Kaiser [4]	1999	Germany	1994-1998	656	8 (1.2)
Mickiene [23]	2002	Lithuania	1998-1999	133	1 (0.8)
Kaiser [5]	2002	Germany	1991-2000	1 500	9 (0.6)
Grygorczuk [6]	2002	Poland	1997-2001	152	1 (0.6)
Zimmermann [7]	2005	Swiss	1999-2004	584	6 (1)
Hansmann [8]	2006	France	1968-2003	64	0 (0)
Logar [9]	2006	Slovenia	2000-2004	448	3 (0.7)
Bogovic [30]	2014	Slovenia	2005-2006	282	2 (0.7)
Czupryna [11]	2011	Poland	1993-2008	621	4 (0.6)
Kriz [31]	2012	Czech Republic	1970-2008	17 053	87 (0.51)
Wahlberg [25]	2006	Finland (Åland)	1959-2005	301	1 (0.33)
Schuler [32]	2014	Schweiz	2005-2006	1055	9 (0.9)
Rezza [26]	2015	Italy	2000-2013	367	2 (0.5)
Fafangel [33]	2017	Slovenia	2009-2013	1190	n.a. (0.75)
Radzišauskienė [12]	2018	Lithuania	2005-2014	712	5 (0.7)
Hellenbrand [14]	2019	Germany	2001-2018	6 063	25 (0.4)
Krawczuk [15]	2020	Poland	2004–2015	601	7 (1.2)
Barp [34]	2020	Italy	2000-2019	148	0 (0)
Children only					
Cizman [35]	1999	Slovenia	1993-1998	133	0 (0)
Lesnicar [16]	2003	Slovenia	1959-2000	371	0 (0)
Zenz [36]	2005	Austria	1980-2003	139	0 (0)
Hansson [24]	2011	Sweden	2003-2008	38	0 (0)
Pazdiora [37]	2012	Czech Republic	1960-2007	410	1 (0.2)
Krbkova [19]	2015	Czech Republic	1993-2012	170	0 (0)
Krawczuk [15]	2020	Poland	2004–2015	68	0 (0)

References for Suppl. Tables S3–5

1. Duniewicz, M.; Mertenová, J.; Moravcová, E.; Jelinková, E.; Holý, M.; Kulková, H.; Doutlik, S. [Central european tick-borne encephalitis from 1969 to 1972 in central bohemia (author's transl)]. *Infection* **1975**, *3*, 223–8, doi:10.1007/bf01642770.

2. Ackermann, R.; Rehse-Küpper, B. [Central European encephalitis in the Federal Republic of Germany (author's transl)]. *Fortschr. Neurol. Psychiatr. Grenzgeb.* **1979**, *47*, 103–22.

3. Kaiser, R. Tick-borne encephalitis in southwestern Germany. *Infection* **1996**, *24*, 398–399, doi:10.1007/BF01716094.

4. Kaiser, R. The clinical and epidemiological profile of tick-borne encephalitis in southern Germany A prospective study of 656 patients. *Brain* **1999**, 2067–2078.

5. Kaiser, R. Tick-borne encephalitis (TBE) in Germany and clinical course of the disease. *Int. J. Med. Microbiol.* **2002**, *291 Suppl*, 58–61, doi:10.1016/S1438-4221(02)80012-1.

6. Grygorczuk, S.; Mierzynska, D.; Zdrodowska, A.; Zajkowska, J.; Pancewicz, S.; Kondrusik, Ma.; Swierzbinska, R.; Pryszmont, J.; Hermanowska-Szpakowicz, T. Tick-borne Encephalitis in North-eastern Poland in 1997-2001: A Retrospective Study. *Scand. J. Infect. Dis.* **2002**, *34*, 904–909, doi:10.1080/0036554021000026979.

7. Zimmermann, H.; Koch, D. Epidemiologie der frühsommer-meningoenzephalitis (FSME) in der Schweiz 1984 bis 2004. *Ther. Umschau* **2005**, *62*, 719–725, doi:10.1024/0040-5930.62.11.719.

8. Hansmann, Y.; Gut, J.P.; Remy, V.; Martinot, M.; Allard Witz, M.; Christmann, D. Tick-borne encephalitis in eastern France. *Scand. J. Infect. Dis.* **2006**, *38*, 520–526, doi:10.1080/00365540600585073.

9. Logar, M.; Bogovič, P.; Cerar, D.; Avšič-Zůpanc, T.; Strle, F. Tick-borne encephalitis in Slovenia from 2000 to 2004: Comparison of the course in adult and elderly patients. *Wien. Klin. Wochenschr.* **2006**, *118*, 702–707, doi:10.1007/s00508-006-0699-6.

10. Karelis, G.; Bormane, A.; Logina, I.; Lucenko, I.; Suna, N.; Krumina, A.; Donaghy, M. Tick-borne encephalitis in Latvia 1973-2009: Epidemiology, clinical features and sequelae. *Eur. J. Neurol.* **2012**, 19, 62-68, 10.1111/j.1468-1331.2011.03434.x.

11. Czupryna, P.; Moniuszko, A.; Pancewicz, S.A.; Grygorczuk, S.; Kondrusik, M.; Zajkowska, J. Tick-borne encephalitis in Poland in years 1993-2008 - epidemiology and clinical presentation. A retrospective study of 687 patients. *Eur. J. Neurol.* **2011**, *18*, 673–679, doi:10.1111/j.1468-1331.2010.03278.x.

12. Radzišauskienė, D.; Žagminas, K.; Ašoklienė, L.; Jasionis, A.; Mameniškienė, R.; Ambrozaitis, A.; Jančorienė, L.; Jatužis, D.; Petraitytė, I.; Mockienė, E. Epidemiological patterns of tick-borne encephalitis in Lithuania and clinical features in adults in the light of the high incidence in recent years: a retrospective study. *Eur. J. Neurol.* **2018**, *25*, 268–274, doi:10.1111/ene.13486.

13. Bogovič, P.; Lotrič-Furlan, S.; Avšič-Županc, T.; Lusa, L.; Strle, F. Factors associated with severity of tick-borne encephalitis: A prospective observational study. *Travel Med. Infect. Dis.* **2018**, doi:10.1016/j.tmaid.2018.10.003.

14. Hellenbrand, W.; Kreusch, T.; Böhmer, M.M.; Wagner-Wiening, C.; Dobler, G.; Wichmann, O.; Altmann, D. Epidemiology of Tick-Borne Encephalitis (TBE) in Germany,

2001–2018. *Pathog. (Basel, Switzerland)* **2019**, *8*, 42, doi:10.3390/pathogens8020042.

15. Krawczuk, K.; Czupryna, P.; Pancewicz, S.; Ołdak, E.; Moniuszko-Malinowska, A. Comparison of tick-borne encephalitis between children and adults—analysis of 669 patients. *J. Neurovirol.* **2020**, *26*, 565–571, doi:10.1007/s13365-020-00856-x.

16. Lešničar, G.; Poljak, M.; Seme, K.; Lešničar, J. Pediatric tick-borne encephalitis in 371 cases from an endemic region in Slovenia, 1959 to 2000. *Pediatr. Infect. Dis. J.* **2003**, *22*, 612–617, doi:10.1097/00006454-200307000-00009.

17. Fritsch, P.; Gruber-Sedlmayr, U.; Pansi, H.; Zöhrer, B.; Mutz, I.; Spork, D.; Zenz, W. Tick-borne encephalitis in Styrian children from 1981 to 2005: A retrospective study and a review of the literature. *Acta Paediatr. Int. J. Paediatr.* **2008**, *97*, 535–538, doi:10.1111/j.1651-2227.2008.00763.x.

18. Stähelin-Massik, J.; Zimmermann, H.; Gnehm, H.E. Tick-Borne Encephalitis in Swiss Children 2000–2004. *Pediatr. Infect. Dis. J.* **2008**, *27*, 555–557, doi:10.1097/INF.0b013e318165c195.

19. Krbková, L.; Štroblová, H.; Bednářová, J. Clinical course and sequelae for tick-borne encephalitis among children in South Moravia (Czech Republic). *Eur. J. Pediatr.* **2015**, *174*, 449–458, doi:10.1007/s00431-014-2401-8.

20. Wahlberg, P.; Saikku, P.; Brummer-Korvenkontio, M. Tick-borne viral encephalitis in Finland. The clinical features of Kumlinge disease during 1959-1987. *J. Intern. Med.* **1989**, *225*, 173–7, doi:10.1111/j.1365-2796.1989.tb00059.x.

21. Holmgren, E.B.; Forsgren, M. Epidemiology of tick-borne encephalitis in Sweden 1956-1989: A study of 1116 cases. *Scand. J. Infect. Dis.* **1990**, *22*, 287–295, doi:10.3109/00365549009027050.

22. Günther, G.; Haglund, M.; Lindquist, L.; Forsgren, M.; Sköldenberg, B. Tick-borne encephalitis in Sweden in relation to aseptic meningo-encephalitis of other etiology: A prospective study of clinical course and outcome. *J. Neurol.* **1997**, *244*, 230–238, doi:10.1007/s004150050077.

23. Mickienė, A.; Laiškonis, A.; Günther, G.; Vene, S.; Lundkvist, Å.; Lindquist, L. Tickborne Encephalitis in an Area of High Endemicity in Lithuania: Disease Severity and Long-Term Prognosis. *Clin. Infect. Dis.* **2002**, *35*, 650–658, doi:10.1086/342059.

24. Hansson, M.E.A.; Orvell, C.; Engman, M.-L.; Wide, K.; Lindquist, L.; Lidefelt, K.-J.; Sundin, M. Tick-borne encephalitis in childhood: rare or missed? *Pediatr. Infect. Dis. J.* **2011**, *30*, 355–7, doi:10.1097/INF.0b013e3181fe3b5a.

25. Wahlberg, P.; Carlsson, S.A.; Granlund, H.; Jansson, C.; Lindén, M.; Nyberg, C.; Nyman, D. TBE in Åland Islands 1959-2005: Kumlinge disease. *Scand. J. Infect. Dis.* **2006**, *38*, 1057–1062, doi:10.1080/00365540600868297.

26. Rezza, G.; Farchi, F.; Pezzotti, P.; Ruscio, M.; Lo Presti, A.; Ciccozzi, M.; Mondardini, V.; Paternoster, C.; Bassetti, M.; Merelli, M.; et al. Tick-borne encephalitis in north-east Italy: a 14-year retrospective study, January 2000 to December 2013. *Eurosurveillance* **2015**, *20*, doi:10.2807/1560-7917.es.2015.20.40.30034.

27. Köck, T.; Stünzner, D.; Freidl, W.; Pierer, K. [Clinical aspects of early summer meningoencephalitis in Styria]. *Nervenarzt* **1992**, *63*, 205–8.

28. Haglund, M.; Forsgren, M.; Lindh, G.; Lindquist, L. A 10-year follow-up study of tick-borne encephalitis in the Stockholm area and a review of the literature: Need for a vaccination strategy. *Scand. J. Infect. Dis.* **1996**, *28*, 217–224.

29. Kaiser, R.; Vollmer, H.; Schmidtke, K.; Rauer, S.; Berger, W.; Gores, D. Verlauf und Prognose der FSME. *Nervenarzt* **1997**, *68*, 324–330, doi:10.1007/s001150050130.

30. Bogovic, P.; Logar, M.; Avsic-Zupanc, T.; Strle, F.; Lotric-Furlan, S. Quantitative evaluation of the severity of acute illness in adult patients with tick-borne encephalitis. *Biomed Res. Int.* **2014**, *2014*, 841027, doi:10.1155/2014/841027.

31. Kriz, B.; Maly, M.; Benes, C.; Daniel, M. Epidemiology of Tick-Borne Encephalitis in the Czech Republic 1970–2008. *Vector-Borne Zoonotic Dis.* **2012**, *12*, 994–999, doi:10.1089/vbz.2011.0900.

32. Schuler, M.; Zimmermann, H.; Altpeter, E.; Heininger, U. Epidemiology of tick-borne encephalitis in Switzerland, 2005 to 2011. *Eurosurveillance* **2014**, *19*, 1–7, doi:10.2807/1560-7917.ES2014.19.13.20756.

33. Fafangel, M.; Cassini, A.; Colzani, E.; Klavs, I.; Grgič Vitek, M.; Učakar, V.; Muehlen, M.; Vudrag, M.; Kraigher, A. Estimating the annual burden of tick-borne encephalitis to inform vaccination policy, Slovenia, 2009 to 2013. *Eurosurveillance* **2017**, *22*, 1–7, doi:10.2807/1560-7917.ES.2017.22.16.30509.

34. Barp, N.; Trentini, A.; Di Nuzzo, M.; Mondardini, V.; Francavilla, E.; Contini, C. Clinical and laboratory findings in tick-borne encephalitis virus infection. *Parasite Epidemiol. Control* **2020**, *10*, e00160, doi:10.1016/j.parepi.2020.e00160.

35. Cizman, M.; Rakar, R.; Zakotnik, B.; Pokorn, M.; Arnez, M. Severe forms of tick-borne encephalitis in children. *Wien. Klin. Wochenschr.* **1999**, *111*, 484–7.

36. Zenz, W.; Pansi, H.; Zoehrer, B.; Mutz, I. Tick-Borne Encephalitis in Children in Styria and. **2005**, *24*, 892–896, doi:10.1097/01.inf.0000180506.76201.43.

37. Pazdiora, P.; Štruncová, V.; Švecová, M. Tick-borne encephalitis in children and adolescents in the Czech Republic between 1960 and 2007. *World J. Pediatr.* **2012**, *8*, 363–366, doi:10.1007/s12519-012-0383-z.

Suppl. Figure S1

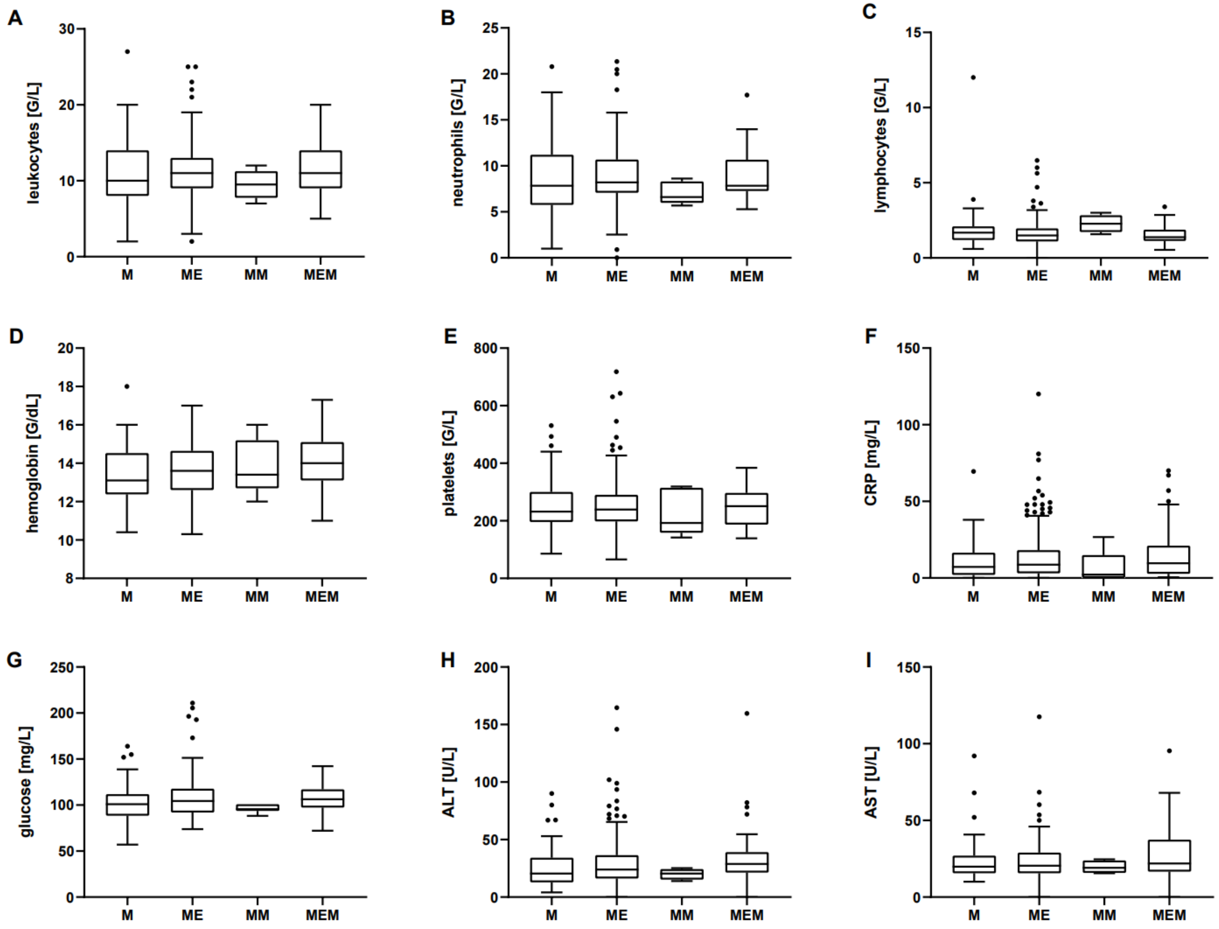

Suppl. Figure S1. Findings in blood on admission.

Suppl. Figure S2

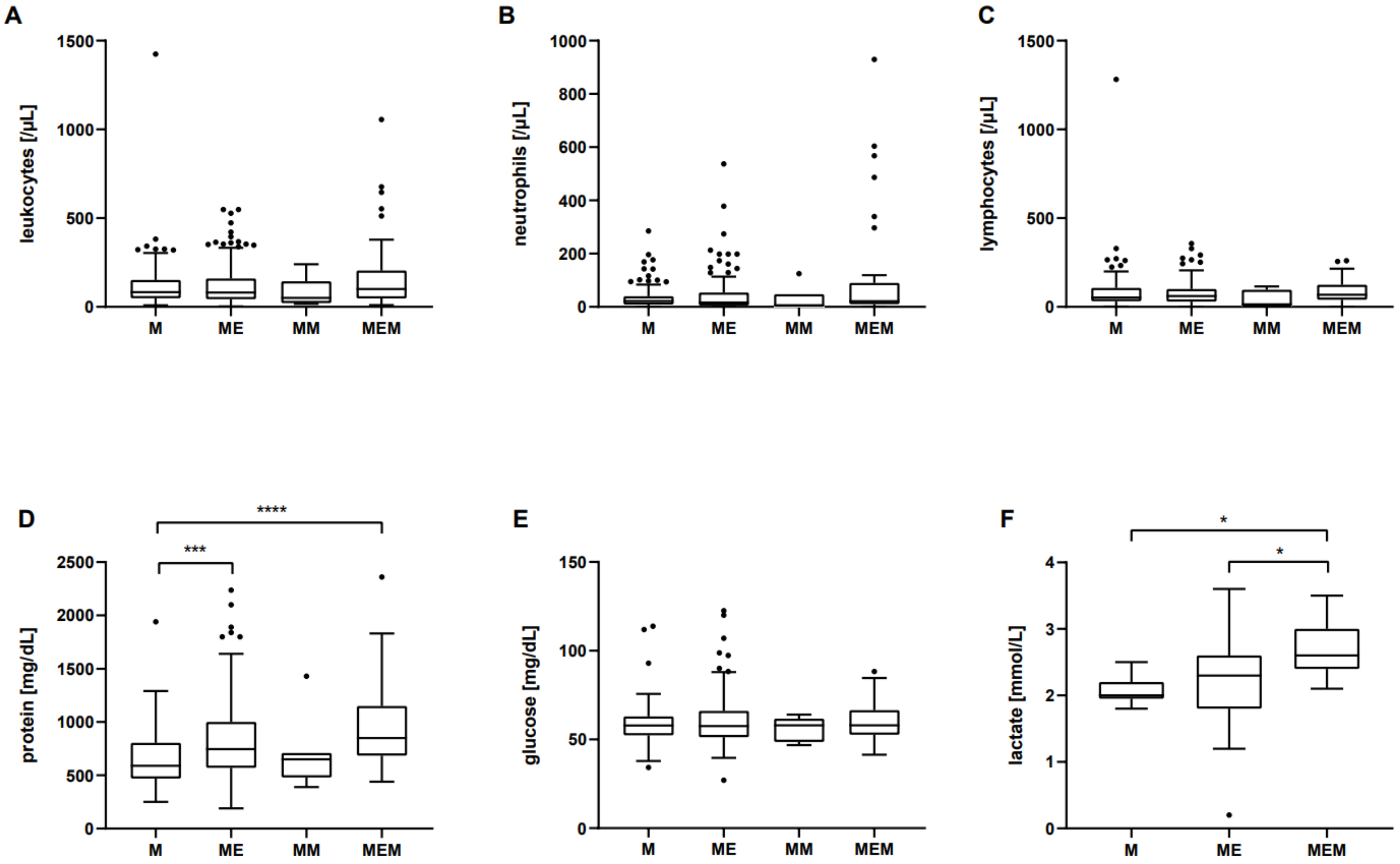

Suppl. Figure S2. Findings in cerebrospinal fluid (CSF). Tukey plot with whiskers. * indicates p=<0.05, ** indicates p=<0.005 and *** indicates p=<0.0005.
